# Regimen‐intensity per count‐recovery and hospitalization index: A new tool to assign regimen intensity for AML

**DOI:** 10.1002/cam4.3311

**Published:** 2020-07-24

**Authors:** Mohamed L. Sorror, Shirali Agarwal, Megan Othus, Ylinne Lynch, Jennifer E. Nyland, Mary‐Elizabeth Percival, Pamela S. Becker, Frederick R. Appelbaum, Elihu H. Estey

**Affiliations:** ^1^ Divisions of Clinical Research Fred Hutchinson Cancer Research Center Seattle WA USA; ^2^ Department of Medicine University of Washington School of Medicine Seattle WA USA; ^3^ Division of Public Health Sciences Fred Hutchinson Cancer Research Center Seattle WA USA; ^4^ Department of Biostatistics University of Washington School of Medicine Seattle WA USA; ^5^ Present address: Department of Neural & Behavioral Sciences Pennsylvania State University State College PA USA

**Keywords:** acute myeloid leukemia, expert opinion, induction therapy, intensity, survey

## Abstract

**Background:**

Low‐intensity regimens have been increasingly used to treat older patients with acute myeloid leukemia (AML). Recent studies, however, suggest older patients can tolerate and potentially benefit from intensive chemotherapeutic regimens. The ability to compare the utility of varying regimen intensities in AML is hindered by the lack of a standardized definition of “regimen intensity.”

**Methods:**

We conducted a survey asking AML physicians which of 38 regimens they would consider intensive vs less‐intensive. Electronic medical records of 592 patients receiving many of these regimens were used to design a model characterizing regimens as intensive vs less‐intensive as identified by ≥75% physician consensus. Variables included frequency and length of hospitalizations, intensive care unit admissions, severe gastrointestinal toxicities, time to nadir, and recovery of neutrophil/platelet count.

**Results:**

Physicians agreed at a rate of 75%‐100% on the assignment of degree of intensity to the majority (n = 28) of these regimens, while the level of agreement was <75% for the remaining 10 regimens (26%). Logistic regression analyses identified number and length of hospitalizations to be significantly associated with intensive regimens and count recovery with less‐intensive regimens. We created the “regimen‐intensity per count‐recovery and hospitalization” (RICH) index with an AUC of 0.87. Independent model validation yielded an AUC of 0.75.

**Conclusions:**

We were able to generate a novel model that defines regimen intensity for many therapies used to treat AML. Results facilitate a future randomized study comparing intensive vs less‐intensive regimens.

## Graphical abstract

Surveyed physicians reached ≥75% concordance on ratings of intensity for 28/38 regimens (74%) used in treatment of acute myeloid leukemia. We developed and validated the “Regimen‐Intensity per Count‐recovery and Hospitalization” (RICH) index, incorporating number and length of hospitalizations and count recovery, to characterize whether a regimen should be considered more‐ or less‐intensive.

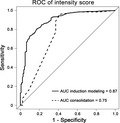

**Key points**

Acute myeloid leukemia (AML) can be treated with either “intensive” or “Less intensive” regimens. We surveyed AML physicians to define which of 38 regimens are intensive or less‐intensive. Physicians agreed on intensity assignment to 28 regimens. Among patients receiving any of these 28 regimens, intensive regimens resulted in more hospitalizations while less‐intensive regimens resulted in slow recovery of blood counts. We incorporated these two factors into a model that helped define the degree of intensity of the remaining 10 regimens that lacked physicians’ consensus. This objective model can be used by clinicians or investigators to determine the intensity of any regimen.

## 1 INTRODUCTION

Survival of patients with newly diagnosed acute myeloid leukemia (AML) is 27% at 5 years.^1^ In patients aged >60 to 65 years and those not considered fit for intensive chemotherapy, average survival is 5 to 10 months,^2^ prompting continued search for more effective, less toxic therapies. There is a recent tendency to treat older patients with less‐intensive therapies assuming they will neither tolerate nor benefit from intensive therapies.^3^, ^4^, ^5^, ^6^ However, the comparative value of regimens of different intensities is uncertain, especially in older and medically infirm patients.^7^, ^8^, ^9^, ^10^, ^11^, ^12^ As a reflection of the uncertainty surrounding best practices in this patient population, ClinicalTrials.gov lists 33 ongoing trials using regimens of varying intensities for initial induction therapy in older AML patients. Comparing the outcomes among regimens of different levels of intensity is hampered by the lack of widely accepted definitions of “intensity.” In an effort to address this deficiency, we conducted the current study taking a sequential approach to define which regimens can be consistently defined as more or less‐intensive. The approach involved (1) surveying physicians experienced in the management of AML to determine which regimens they would consider, with a high degree of consensus, as more‐ or less‐intensive, (2) examining covariates that are indicative of toxicity and response and associated with the physicians’ consensus about intensive vs less‐intensive regimens, and (3) developing a multivariate model incorporating these covariates that can be used to define intensities (a) of the remaining regimens for which there was less consensus, and (b) of future regimens.

## 2 METHODS

We followed the Enhancing the QUAlity and Transparency of Health Research (EQUATOR) reporting guidelines that employ the Transparent Reporting of a Multivariable Prediction Model for Individual Prognosis Or Diagnosis (TRIPOD) criteria.^13^, ^14^ The EQUATOR network is an international initiative aimed to improve the reliability and value of published health research literature by promoting transparent and accurate reporting. The EQUATOR network has created a set of specific guidelines for different types of study reporting. For prognostic studies, the EQUATOR network has created the TRIPOD criteria.

The TRIPOD criteria were developed to ensure full and clear reporting of information on all aspects of a prediction model to enable proper assessment of risk of bias and potential usefulness of that model. The TRIPOD includes a set of 22 recommendations that were developed through a series of surveys and meetings among experts. The TRIPOD 22 checklist items were deemed essential for transparent reporting of a prediction model study, and they cover details such as sources of data, model performance, study limitations, and model implications.

### 2.1 Survey design

Our survey principally inquired about regimens used to treat 592 patients treated consecutively at our institution between 2008 and 2017 since we would be able to correlate survey respondents’ impressions of these regimens’ intensity with toxicity and response data derived from patients given the regimens. We also asked about other regimens used in clinical trials currently listed as active at ClinicalTrials.gov, or discussed in presentations at the 2017 American Society of Hematology meeting. We combined regimens into major categories (eg high‐dose cytarabine containing) and eliminated rarely used regimens or agents. This resulted in 38 agents/regimens available to be included in the survey.

The study's first and last senior authors (MLS and EE) identified 120 academic AML‐treating physicians associated with organizations such as the Acute Leukemia French Association; Alliance Cooperative Group; Eastern Cooperative Oncology Group; European LeukemiaNet; Hemato‐Oncology Foundation for Adults in the Netherlands; Medical Research Council; National Comprehensive Cancer Network; and SWOG. We randomly selected 55 of these physicians and sent them invitations to complete a 3 to 5‐minute online survey. The survey was open for 7 weeks, with reminders sent weekly. Experts were asked to classify each agent or regimen as “less‐intensive,” “intensive,” or “uncertain.” Two additional questions asked respondents to name “other regimens not mentioned in the survey” and indicate “years of experience in Oncology practice:” “<5 years,” “5‐10 years,” “10‐15 years,” “15‐20 years,” “20‐25 years,” and “>25 years.”

We suggested therapies be classified as intensive if intended to achieve complete remission (CR) without measurable residual disease,^15^ often after one cycle of induction but at the potential expense of increased toxicity. Therapies classified as less‐intensive would have the putative advantage of reduced toxicity and might aim to achieve lesser responses such as complete response with incomplete count recovery (CRi) or “marrow leukemia free state” based on the assumption that these responses, although often requiring more than one cycle, are associated with longer survival than no response.^16^

A regimen was considered more or less‐intensive if there was ≥75% agreement as to its classification among survey participants and was considered of uncertain intensity if there was <75% concordance.

### 2.2 Covariates associated with regimen intensity

We next examined covariates characteristic of regimens considered more or less‐intensive by the respondents to our survey. These regimens were administered between 1st January 2008 and 16th June 2017 at Fred Hutchinson Cancer Research Center. Information regarding the following was collected: the number and cumulative length of hospitalizations within the first 35 days after treatment; the number of intensive care unit admissions within the first 35 days after treatment; grade III‐IV gastrointestinal toxicities (per the National Cancer Institute Common Toxicity Criteria) within the first 35 days after treatment; time to nadir of absolute neutrophil count (ANC) and of platelet count; time from nadir of ANC or platelet count until return of ANC and platelet count to >1000 and 100 000, respectively; and response to therapy (no response vs response but with minimal residual disease [MRD] vs no MRD).

### 2.3 Statistical methods

#### 2.3.1 Survey analyses

Kappa statistics were used to evaluate inter‐rater agreement in surveys.^17^, ^18^ Kappa statistics adjust for the degree of agreement that would be expected to occur by chance, and are therefore, more appropriate than Pearson's product moment, Spearman's correlation, or percent agreement.^19^ We used Fleiss’ Kappa to adjust for multiple raters.^18^ The Landis scale (range −1 to 1) was used to interpret kappa statistics where values <0 indicate no agreement; 0.0 to 0.20, slight; 0.21 to 0.40, fair; 0.40 to 0.60, moderate; 0.61 to 0.80, substantial; and 0.81 to 1.00, almost perfect agreement.^20^ Wilcoxon Rank‐Sum tests were used to compare agreement on definitions of intensity with years of physician experience (≤25 years vs >25 years).

#### 2.3.2 Model development and validation

The covariates found characteristic of regimen intensity were incorporated into a multivariate logistic regression model intended to distinguish induction regimens (given to a training group of 592 patients) that ≥75% of respondents felt were “intensive” from those that ≥75% of respondents felt were “less‐intensive.” Our intent was to use the model to classify regimens where there was <75% agreement per the survey results. Weights for the model were created by converting the (base e) logarithms of the odds ratios into scores. Sensitivity and specificity were used to evaluate a model score cutoff to define regimen intensity. The area under the curve (AUC) of the receiver operating characteristic (ROC) was calculated to evaluate model performance within the training cohort using fivefold cross‐validation. This was done using the bootstrapping method, which is a statistical method that repeatedly takes samples from the original data set and repeats the same analysis on these bootstrap samples. The variation across the bootstrap sample provides a good estimate of the variation in the original estimate. We validated the model using data from 288 patients receiving postremission consolidation therapies.

## 3 RESULTS

Thirty‐three of 55 (64%) physicians returned surveys. One of these 33 physicians had less than 5 years’ experience, 8 had 5 to 15 years' experience, 8 had 20‐25 years’ experience, and 15 had more than 25 years of experience.

The survey included 6 single agents and 32 combination regimens. Among these 38 agents/regimens, surveyed physicians agreed (≥75% agreement) on a designation of either “intensive” or “less‐intensive” for 28 (74%) and disagreed (<75% agreement) for 10 regimens (26%; 95% CI 14%‐43%), with an overall kappa statistic estimate of moderate value (0.54). Of the 28 regimens that were agreed upon (≥75% agreement), 18 were considered “intensive” and 10 “less‐intensive” (Table 1).

**TABLE 1:** Summary of expert survey results

Regimen^a^	Total responses (n)	Less‐intensive (%)	Intensive (%)	Undecided (%)
Consensus: less‐intensive				
Azacitidine or decitabine alone	33	100	0	0
Azacitidine or decitabine + lenalidomide	32	81	16	3
Azacitidine or decitabine + midostaurin	33	97	0	3
Azacitidine or decitabine + venetoclax (BCL2 inhibitor)	33	76	12	12
Azacitidine or decitabine + vorinostat	32	94	3	3
Azacitidine or decitabine + any other single‐targeted novel agent	32	78	0	22
Guadecitabine (DNMT inhibitor) ± other	33	79	3	18
Low‐dose Ara‐C (LDAC) alone	33	100	0	0
Low‐dose Ara‐C (LDAC) + venetoclax (BCL2 inhibitor)	33	76	9	15
Low‐dose Ara‐C (LDAC) + any other single‐targeted novel agent	33	82	0	18
Consensus: intensive				
Cytarabine (100 mg/m^2^/dose) + mitoxantrone	33	3	97	0
Cytarabine (≥1 g/m^2^/dose) alone including HiDAC (high‐dose cytarabine)	33	3	94	3
Cytarabine (≥1 g/m^2^/dose) + idarubicin	33	0	100	0
Cytarabine (≥1 g/m^2^/dose) + midostaurin	31	6	87	7
Cytarabine (≥1 g/m^2^/dose) + purine analog (fludarabine, cladribine, or clofarabine) + GCSF	33	3	94	3
Cytarabine (≥1 g/m^2^/dose) + mitoxantrone + etoposide [MEC] ± other	31	0	97	3
Cytarabine (≥1 g/m^2^/dose) + any other single‐targeted novel agent	33	0	88	12
Cytarabine + idarubicin or daunorubicin '7 + 3' alone	33	0	100	0
Cytarabine + idarubicin or daunorubicin '7 + 3' + cladribine	31	0	97	3
Cytarabine + idarubicin or daunorubicin '7 + 3' + decitabine	32	0	97	3
Cytarabine + idarubicin or daunorubicin '7 + 3' + etoposide	32	0	100	0
Cytarabine + idarubicin or daunorubicin '7 + 3' + gemtuzumab ozogamicin (GO)	33	0	100	0
Cytarabine + idarubicin or daunorubicin '7 + 3' + midostaurin	33	0	97	3
Cytarabine + idarubicin or daunorubicin '7 + 3' + sorafenib	33	0	97	3
Cytarabine + idarubicin or daunorubicin '7 + 3' + any other single‐targeted novel agent	33	0	88	12
CPX‐351 (liposomal combination of cytarabine + daunorubicin)	32	3	97	0
Gemtuzumab ozogamicin (GO) + idarubicin	33	6	91	3
IAP (ifosfamide, adriamycin, cisplatin)	33	3	79	18
No consensus				
AMG232 (MDM2 inhibitor) ± trametinib	31	58	0	42
BET inhibitor	32	66	0	34
Clofarabine	33	24	73	3
Cytarabine (100 mg/m^2^/dose) + any other single‐targeted novel agent	33	27	61	12
Gemtuzumab ozogamicin (GO) alone	33	67	33	0
Gemtuzumab ozogamicin (GO) + vorinostat	31	55	32	13
Azacitidine or decitabine + gemtuzumab ozogamicin (GO)	33	67	33	0
Low‐dose Ara‐C (LDAC) + gemtuzumab ozogamicin (GO)	33	73	27	0
Low‐dose Ara‐C (LDAC) + aclarubicin	33	45	36	21
Sorafenib + vorinostat +bortezomib	33	64	9	25

Note

Agents/regimens excluded from the survey because they were given to relatively few patients were H‐CVAD (cyclophosphamide + vincristine + doxorubicin + dexamethasone), Ibrutinib, and SGN‐CD22a.

^a^ A regimen was considered intensive if there was ≥75% agreement on this designation and likewise for non‐intensive. Regimen intensity was considered undecided if agreement was <75%.

Examples of intensive regimens acquiring ≥75% agreement include the standard “7 + 3” and cytarabine at doses of at least 1 g/m^2^. Examples of “less‐intensive” regimens acquiring ≥75% agreement were those using azacitidine or decitabine alone, azacitidine or decitabine combined with agents such as lenalidomide or midostaurin, and low‐dose cytarabine (Ara‐C, 20 mg/m^2^). Undecided regimens (<75% agreement) included gemtuzumab ozogamicin (GO) alone; GO combined with either azacitidine, decitabine, or low‐dose Ara‐C; single agent clofarabine; and cytarabine at a dose of 100 mg/m^2^ combined with any other single‐targeted novel agent.

The number of years of experience was not well correlated with the level of agreement on intensity of a regimen. The rates of agreement were 82% vs 76% (*P* = .057) among those with ≤25 years vs those with >25 years of experience.

### 3.1 Model development and validation

Patients receiving the 28 regimens with a high rate of agreement were characterized with respect to the above‐noted covariates to derive a model to define intensity among patients receiving the other 10 (uncertain) regimens. In logistic regression analyses, covariates found to be associated (based on odds ratios with *P* < .01) with characterization of regimens as “intensive” rather than “less‐intensive” were: having at least one hospitalization within the first 35 days and hospitalization stays of >15 days (Table 2). Covariates associated with ≥75% agreement that a regimen was “less‐intensive” were: lack of platelet recovery or neutrophil recovery by day 28.

**TABLE 2:** Logistic regression model to identify variables defining regimen intensity^a^

	OR^b^	95% CI	*P*‐value
1 hospitalization (ref = No hospitalization)	14.16	(5.79, 34.66)	<.001
2 hospitalizations (ref = No hospitalization)	22.05	(8.45, 57.56)	<.001
3 to 4 hospitalizations (ref = No hospitalization)	51.73	(13.34, 200.63)	<.001
Days hospitalized 15 to 28 d (ref = Days hospitalized 0 to 14 d)	2.56	(1.37, 4.8)	.0033
Days hospitalized ≥ 28 d (ref = Days hospitalized 0 to 14 d)	4.96	(2.39, 10.29)	<.001
Platelet recovery (100 000) in > 28 d (ref = Platelet recovery (100 000) in ≤28 d)	0.67	(0.29, 1.56)	.35
No platelet (100 000) recovery (ref = Platelet recovery (100 000) in ≤28 d)	0.31	(0.14, 0.67)	.003
ANC recovery (1000) in > 28 d (ref = ANC recovery (1000) in ≤28 d)	1.11	(0.53, 2.31)	.79
No ANC recovery (1000) (ref = ANC recovery (1000) in ≤28 d)	0.28	(0.13, 0.6)	.0011

Abbrevaitions: ANC, absolute neutrophil count; CI, confidence interval; OR, odds ratio.

^a^ Only significant nondemographic variables are presented here. These variables are to be used to build the RICH model.

^b^ OR > 1 indicates increased odds that a patient was treated with a regimen that was identified as intensive by ≥75% of respondents.

While age had a statistically significant association with the choice of less‐intensive therapies (Odds Ratio [OR]: 0.86, 95% CI: 0.83‐0.9, and *P* < .001), other demographics such as gender and race were not statistically associated with the intensity of regimens (data not shown).

On the contrary, MRD after first cycle (OR: 0.5, 95% CI: 0.2‐1.23, and *P* = .13); no response after first cycle (OR: 0.47, 95% CI: 0.21‐1.06, and *P* = .07); at least one ICU admission (OR: 1.59, 95% CI: 0.76‐3.29, and *P* = .22); and GI toxicities (OR: 1.0, 95% CI: 0.53‐1.88, and *P* = .99) did not have an association with the intensity of regimens.

**FIGURE 1:**
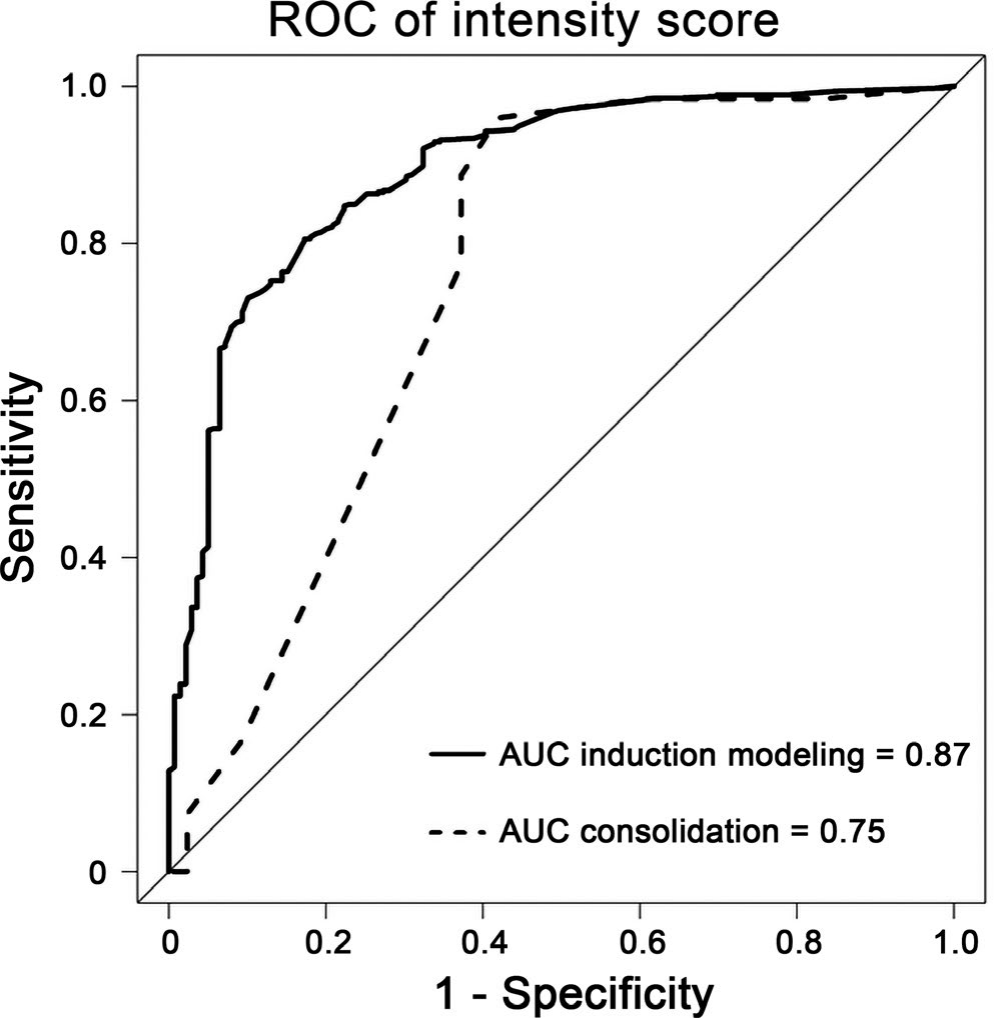
Receiver operating characteristic curve for assignment of regimen intensity. The RICH model had area under the curve (AUC) of 0.87 among patients receiving induction chemotherapy and AUC of 0.75 among those receiving consolidation therapy

We next converted the (base e) logarithms of the odds ratios into scores to create the Regimen‐Intensity per Count‐recovery and Hospitalization (RICH) model (Table 3). The cross‐validated bootstrap‐corrected estimate of AUC was 0.87 (Figure 1). A RICH score of 2.5 or higher was associated with both high sensitivity (85%) and specificity (75%) to predict an “intensive” regimen (Table 4). To further validate and confirm generalizability of the model, we applied the RICH index to a cohort of patients (n = 288) receiving postremission consolidation therapies. The RICH index classified regimen intensity in the validation cohort with an AUC of 0.75 (Figure 1). Among the regimens used to treat these 288 patients, 27 were designated as intensive and 14 as less‐intensive.

**TABLE 3:** RICH index score assignment based on rounding (base e) logarithms of odds ratios

	Score
Number of hospitalizations	
No hospitalization	0
1 hospitalization	3
2 hospitalizations	3
3 to 4 hospitalizations	4
Days hospitalized	
0 to 14 d	0
15 to 28 d	1
≥28 d	2
Number of platelet recoveries (100 000) within	
≤28 d	0
>28 d	0
No platelet recovery	−1
ANC recoveries (1000) within	
≤28 d	0
>28 d	0
No ANC recovery (1000)	−1

**TABLE 4:** Sensitivity and specificity of intensity scoring thresholds^a^

Threshold	Sensitivity (%)	Specificity (%)
−Inf	100	0
−1.5	99	15
−0.5	99	29
0.5	98	40
1.5	94	59
2.5	85	75
3.5	60	94
4.5	27	99
5.5	1	100
Inf	0	100

^a^ Score cutoff of 2.5 was associated with the highest combined sensitivity and specificity.

Table 5 depicts regimens for which there was ≥75% agreement of either “intensive” or “less‐intensive.” The former included the standard “7 + 3” and doses of at least 1 g/m^2^ of cytarabine with or without other agents. Their median RICH scores were 4 and 3, respectively. In contrast, regimens for which there was ≥75% agreement on them being less‐intensive regimens, median RICH scores were −1 for regimens such as azacitidine or decitabine with or without other agents, and a score of 1 for low‐dose Ara‐C with or without other agents.

**TABLE 5:** RICH scores assigned to specific regimens and how the designation of intensity is made based on cutoff scores of >2.5

Regimen/agent	Patients	Median RICH score	Designation of intensity
Classic “7 + 3”	75	4	Intensive
FLAG	7	3	Intensive
GCLAM ± others	151	3	Intensive
IAP	35	4	Intensive
Azacitidine or decitabine ± others	21	−1	Less‐intensive
LDAC ± others	9	1	Less‐intensive
Gemtuzumab ozogamicin ± others	30	0.5	Less‐intensive
Clofarabine ± others	15	1	Less‐intensive

On the contrary, controversial regimens with <75% agreement per survey responses included GO with or without other agents and clofarabine with or without other agents. Applying RICH model on these regimens, median RICH scores were 0.5 and 1.0, respectively, suggesting a lower intensity designation for these regimens.

## 4 DISCUSSION

There are many treatment options for AML, and efforts continue to develop agents that target‐specific molecular features of AML. One factor typically considered in the choice of treatment regimen is its categorization as of either intensive or less‐intensive. This presupposes agreement as to which category a given regimen belongs. We believe this is the first attempt to assess the extent of concordance regarding categorization of regimen intensity. Based on the kappa statistic, the 35 surveyed physicians reached a reasonable consensus about the degree of intensity among the majority of AML regimens (28 out of 38 regimens). Years of physician experience had a statistically nonsignificant impact on the categorization of regimen intensity.

Although our data indicates general consensus among AML physicians regarding the degree of intensity of a given regimen, our results suggest such designation is considerably short of uniform. Thus, for 10 out of 38 regimens there was less than 75% agreement in intensity designation. Although the choice of 75% as a criterion of concordance is arbitrary, it is midway between complete agreement and agreement only at the 50% level of a coin‐flip, and thus, seemed like a reasonable choice. As such, our data suggest need for a more objective and consistent approach to assignment of regimen intensity. Here, we found that frequency of hospital admission, length of hospitalization, and blood count recovery can be used to develop an index (“RICH”) that can characterize with high sensitivity and specificity whether a regimen should be considered intensive or less‐intensive when physicians might only agree at the approximate 75% level considered here. The index was validated by two distinctive approaches: (a) via fivefold cross‐validation within the population given induction therapies and (b) via external cross‐validation in patients receiving consolidation therapies. The capacity of discrimination was strong with AUC estimates of 0.87 and 0.75, respectively. The successful cross‐validation in a cohort receiving consolidation therapies indicates the potentially wide applicability and generalizability of the RICH model for all therapies used to treat AML.

Not surprisingly, older age was associated with a greater tendency to receive less‐intensive therapies. However, our goal was to determine regimen intensity based on outcomes, such as toxicities, hospitalizations, and response after receiving that regimen. Therefore, age was not included among the variables to build the RICH model. Nevertheless, when we included age in the multivariate model, results of associations between count recover and hospitalization with regimen intensity did not change.

### 4.1 Limitations

Although we had no missing data regarding the components of the RICH model, as with any retrospective analysis, we could potentially have failed to capture important but unrecognized, and thus, unrecorded data. Second, we used number and cumulative length of hospitalizations as one of the criteria to define regimen intensity, but centers may have different policies for hospitalization. Yet, we believe all tertiary cancer centers delivering AML‐like therapy agree upon admission to closely monitor patients that are expected to have significant toxicities from intensive regimens and to keep them hospitalized until counts are recovered or until 4 weeks have elapsed, whichever comes first. Likewise, one of the main reasons for selecting less‐intensive therapies is to avoid planned patient admission given expectations of lessened toxicities. These notions were actually confirmed by the 33 physicians who agreed on 28 regimens to be either intensive or less‐intensive as these physicians were from different organizations. In addition, we built the model on criteria associated with regimens grouped by the physicians’ consensus by their level of intensity. Hence, frequency and durations of hospitalizations can be a good surrogate for regimen‐induced toxicities rather than simply a reflection of hospital policies. It is important in the future to validate the RICH model at other institutions.

Inclusion in the RICH model of platelet and ANC recovery data add objectivity to the model, assuming data on blood counts are obtained at similar intervals at different centers; we believe this is a fair assumption. Finally, our physician survey that led us to consider induction regimens as more intensive, less‐intensive, or uncertain might have produced different results if given to different physicians or if all 55 physicians to whom we sent surveys had responded, a limitation that is not unique to our survey.

### 4.2 Model applications and benefits

With the introduction of many less‐intensive regimens, exemplified by venetoclax + azacitidine or decitabine, the issue of whether a given regimen is intensive or less‐intensive has come to the forefront. Although, as shown here, there is typically agreement as to intensity designation, we found there was <75% agreement in 10 out of 38 (26%; 95% CI 14%‐43%) cases. Disagreement may become more common as newer regimens are introduced or as new less‐intensive agents are combined with one another. In such cases, the RICH model can be used to objectively define regimen intensity, allowing for an accurate comparison of trial results with regimens of similar intensities where consensus regarding intensity is elusive. We plan to use the RICH model to stratify regimens for a future randomized trial comparing intensive vs less‐intensive regimens. Although today this is a relatively simple undertaking, as for example a trial randomizing between 7 + 3 and decitabine, delineation of regimens as intensive or less‐intensive may become more difficult than is the case with 7 + 3 or decitabine. If so, tools such as the RICH index might be of particular value.

The RICH model could also guide less experienced physicians in assessing whether regimens are intensive or less‐intensive when making decisions in the clinic. Finally, this analysis could be used as a template to define regimen intensities for other malignancies or for other interventions in AML, such as conditioning regimens of different intensities used before allogeneic hematopoietic cell transplantation.

## 5 CONCLUSIONS

While AML physicians in general agree on the characterization of regimen intensity for AML therapies, a number of newly developed agents/regimens lack this type of consensus. To address this gap of knowledge, we were able to develop and validate a novel model, RICH index, that can reliably stratify intensities among less common or future regimen, with benefits as described above.

## CONFLICT IN INTEREST

Dr Becker receives research support from Abbvie, Amgen, BMS, Glycomimetics, JW Pharmaceuticals, Novartis, Pfizer, Trovogene, is a speaker for France Foundation (CME); and a consultant for CVS Caremark and McKesson. Dr Appelbaum reports personal fees from Adaptive Biotechnologies, personal fees from Amgen, personal fees from Celator, outside the submitted work. Dr Sorror served on advisory board and received personal fees from Jazz Pharmaceuticals, outside the submitted work. Dr Sorror received clinical trial support from Celgene, outside the submitted work.

## AUTHOR CONTRIBUTIONS

MLS, MO, and EHE conceived and designed the experiments; MLS, SA, YL, JEN, and EHE provided for the collection and assembly of data; MLS, SA, MO, JEN, YL, MEP, PSB, FRA, and EHE participated in data analysis and interpretation. All authors participated in writing of the manuscript and approved of the final draft.

## ETHICAL APPROVAL

The study was approved by an Institutional Review Board.

## Data Availability

The data that support the findings of this study are available from the corresponding author upon reasonable request and upon agreement from all coauthors.
